# “A Good Personal Scientific Relationship”: Philip Morris Scientists and the Chulabhorn Research Institute, Bangkok

**DOI:** 10.1371/journal.pmed.0050238

**Published:** 2008-12-23

**Authors:** Ross MacKenzie, Jeff Collin

**Affiliations:** 1 School of Public Health, University of Sydney, Sydney, New South Wales, Australia; 2 Centre for International Public Health Policy, University of Edinburgh, Edinburgh, Scotland; University of California San Francisco, United States of America

## Abstract

**Background:**

This paper examines the efforts of consultants affiliated with Philip Morris (PM), the world's leading transnational tobacco corporation, to influence scientific research and training in Thailand via the Chulabhorn Research Institute (CRI). A leading Southeast Asian institute for environmental health science, the CRI is headed by Professor Dr. Her Royal Highness Princess Chulabhorn, the daughter of the King of Thailand, and it has assumed international significance via its designation as a World Health Organization (WHO) Collaborating Centre in December 2005.

**Methods and Findings:**

This paper analyses previously confidential tobacco industry documents that were made publicly available following litigation in the United States. PM documents reveal that ostensibly independent overseas scientists, now identified as industry consultants, were able to gain access to the Thai scientific community. Most significantly, PM scientist Roger Walk has established close connections with the CRI. Documents indicate that Walk was able to use such links to influence the study and teaching of environmental toxicology in the institute and to develop relations with key officials and local scientists so as to advance the interests of PM within Thailand and across Asia. While sensitivities surrounding royal patronage of the CRI make public criticism extremely difficult, indications of ongoing involvement by tobacco industry consultants suggest the need for detailed scrutiny of such relationships.

**Conclusions:**

The establishment of close links with the CRI advances industry strategies to influence scientific research and debate around tobacco and health, particularly regarding secondhand smoke, to link with academic institutions, and to build relationships with national elites. Such strategies assume particular significance in the national and regional contexts presented here amid the globalisation of the tobacco pandemic. From an international perspective, particular concern is raised by the CRI's recently awarded status as a WHO Collaborating Centre. Since the network of WHO Collaborating Centres rests on the principle of “using national institutions for international purposes,” the documents presented below suggest that more rigorous safeguards are required to ensure that such use advances public health goals rather than the objectives of transnational corporations.

## Abstract 2

Jeff Collin and Ross MacKenzie analyze tobacco industry documents and find that Philip Morris consultants were able to gain access to a Thai research institute that is a WHO Collaborating Centre.

## Editors' Summary

**Background.:**

Tobacco use kills 5.4 million people a year (one person every six seconds) and accounts for one in ten adult deaths worldwide. Globally, the use of tobacco is on the rise, especially in developing countries, which have become a major target for tobacco industry marketing. The tobacco industry has worked hard to try and influence public perceptions about the risks of smoking and the risk of inhaling secondhand smoke (passive smoking). The industry has used a variety of tactics to downplay the health hazards of smoking or inhaling secondhand smoke—two examples are publishing articles casting doubts about the health hazards of tobacco and funding research that is biased toward giving pro-industry results. Another tactic is for tobacco industry consultants to try and gain entry to universities and other academic centers to see if they can influence research and teaching activities.

**Why Was This Study Done?:**

The researchers were concerned that consultants from the tobacco company Philip Morris had gained access to an academic research center in Thailand called the Chulabhorn Research Institute (CRI). The CRI is an internationally renowned teaching institution for a variety of scientific disciplines, including environmental toxicology (the study of how chemicals in the environment, such as tobacco smoke, can affect human health), biomedicine, and biotechnology. The institute has secured funding from the Thai government, the Association of Southeast Nations and the United Nations Development Programme. In 2005 the institute's environmental toxicology unit was designated a World Health Organization (WHO) Collaborating Centre. WHO Collaborating Centres are “institutions such as research institutes, parts of universities or academies, that are designated by the Director-General of the WHO to carry out activities in support of the WHO's programs” (http://www.who.int/collaboratingcentres/en/). The researchers were concerned that Philip Morris consultants had been able to develop relationships with the CRI to help advance the company's interests.

**What Did the Researchers Do and Find?:**

The researchers analyzed previously confidential tobacco industry documents that were made publicly available online following litigation in the United States. They searched two online collections of industry documents—the Legacy Tobacco Documents Library and Tobacco Documents Online—as well as the online collections operated by US-based tobacco companies. They found that consultants to Philip Morris were able to gain access to the scientific community in Thailand. A Philip Morris scientist named Roger Walk was able to establish close connections to the CRI, and he used these connections to influence research and teaching activities at the CRI on environmental toxicology. Walk was also able to build relationships with government officials and scientists in Thailand to help advance the interests of Philip Morris in the country and across Asia.

**What Do these Findings Mean?:**

This study provides evidence that the tobacco industry has established close links with a research institute in Thailand that collaborates with the WHO, and has been able to influence the institute's teaching curriculum and research. Such links are of great concern to the public health community, which is working hard to reduce deaths and disease due to tobacco. These links raise the possibility that the tobacco industry is managing to influence medical research and teaching at academic institutions. The WHO has stated that a firewall is in place between itself and the tobacco industry—but the study authors argue, based on their findings, that “this firewall is not impenetrable.” The study findings, they conclude, highlight a challenge posed to international tobacco control efforts, especially with respect to Article 5.3 of an international treaty called the WHO Framework Convention on Tobacco Control; Article 5.3 addresses the need to protect public health policies from the vested interests of the tobacco industry. The authors say that better safeguards must be put in place to prevent tobacco companies from thwarting public health goals.

**Additional Information.:**

Please access these Web sites via the online version of this summary at http://dx.doi.org/10.1371/journal.pmed.0050238.

- The Legacy Tobacco Documents Library contains over 9.7 million documents created by tobacco companies
- Tobacco Documents Online contains over 4 million tobacco industry documents
- Over 900 WHO Collaborating Centres are at work in 99 Member States on many health disciplines
- The WHO held an inquiry in 2000 into possible tobacco industry influence over the organization (and over other UN agencies), and has published its recommendations in response to this inquiry
- The WHO Framework Convention on Tobacco Control is an international treaty on controlling tobacco

## Introduction

Release of previously confidential corporate documents has demonstrated the extensive and longstanding efforts by tobacco companies to influence scientific understanding and public perceptions of the health impacts of smoking [1–3]. Tobacco companies were particularly concerned about health risks associated with exposure to passive or secondhand smoke, what the industry has termed “environmental tobacco smoke” (ETS), and undertook broad campaigns to confound public and official knowledge of those risks.

Strategies designed to ensure the release of research findings favourable to the tobacco industry and raise doubts regarding findings of independent science [4–9] included funding of research at seemingly independent organisations such as the International Life Sciences Institute [10,11]; acquisition of established research institutions, as in the case of purchase by Philip Morris (PM) of the Institut für Industrielle und Biologische Forschung GmbH (INBIFO) [12]; initiatives to undermine World Health Organization (WHO) tobacco control programmes and agencies [13], notably the International Agency for Research on Cancer (IARC) [14]; and recruitment of ostensibly independent scientists and academics.

PM's “global strategy on environmental tobacco smoke” [15] stressed the particular importance of establishing regional teams of “independent” experts overseen by a “coordinating scientist and American lawyers, to review scientific literature or carry out work on ETS to the keep the controversy alive” [15]. The “Whitecoat Project” recruited scientists and academics, whose industry affiliations were unpublicised, to support efforts to resist and repeal existing smoking restrictions by reversing the “scientific and popular misconception that ETS is harmful” [16].

Toxicologists were of considerable importance to the ETS project, particularly those with expertise in the field of environmental toxins and their physiological, biochemical, and epidemiological impacts. Their special value lay in their ability to authoritatively dispute independent scientific findings on ETS by suggesting the evidence was inconclusive [17]. A 1987 discussion of the ETS programme in PM's Eastern Europe, Middle East & Africa region stressed the need to recruit and educate “consultants(s)” to operate as “credible third-party spokesmen in environmental toxicology and ETS” [18]. PM also hired a Washington, D. C.-based environmental health consulting firm to identify potential consultant scientists for Scandinavia based on their expertise in toxicology and indoor air quality that same year [19].

### The ETS Programme in Asia

Regional efforts to manipulate the conduct and dissemination of scientific research in Asia have been driven by the tobacco industry's need to slow the spread of effective regulation across a region that is critical both to its future profitability and to the trajectory of the tobacco pandemic. Previous research has shown that the Asian component of this project entailed use of consultants with specialities in chemistry, epidemiology, occupational health, toxicology, and building science [20], to publish articles, to lobby policy makers, and to monitor scientific meetings [19]. Their work placed a premium on emphasising air quality issues specific to the region including heat, humidity, incense smoke, and indoor cooking on coal burners [21]. These efforts provided, according to PM, “opportunities for communicating to the public and policy makers the relative and minor contribution of ETS to the indoor environment within the context of the serious indoor and outdoor air quality problems encountered in this region” [22].

### Scientific Initiatives in Thailand

Thailand has long been recognised as a “leader among developing countries in getting gold-standard tobacco control policies in place” [23] and a key influence in the regional development of tobacco regulation [24–27]. The country's comprehensive 1992 tobacco control legislation package has been supplemented by enactment of laws requiring pictorial health warnings on cigarette packs [28] and a ban on cigarette displays at point of sale in retail outlets [29]. Further measures include a series of tobacco tax increases to the current rate of 75% [30]; and the establishment in 2001 of the Thai Health Promotion Foundation, a state agency supporting public health promotion funded by a dedicated tax on cigarettes and alcohol [31].

This level of regulation and the country's comparatively advanced regional research capacity are reflected in industry concern to build close links with local scientists and institutions. Previous studies describing the Asian ETS Consultants Program, the regional manifestation of the ETS Consultants Program, provide limited discussion of its specific impact in Thailand [19,21]. Our analysis of tobacco industry documents indicates that contacts with Thai scientists, academics, and government officials were established by PM scientists and consultants from the late 1980s.

Of particular significance to the Thai situation is evidence documenting a longstanding relationship between PM scientists and the Chulabhorn Research Institute (CRI) in Bangkok, a leading Southeast Asian scientific research and teaching institution for medicinal chemistry, environmental toxicology, biomedicine, and biotechnology [32]. The institute has established an impressive international profile, and has secured funding from the Thai government [33], the Association of Southeast Asian Nations [34] and the United Nations Development Programme [35]. Its regional significance is reflected in the international training courses it has run in conjunction with WHO, including the IARC [36–38], and with the International Union Against Cancer [39]. Its regional capacity-building project on environmental and industrial toxicology has included seminars in Vietnam, Indonesia, and Malaysia [40–42], funded by the Thai government and the United Nations Development Programme [33]; and a series of Association of Southeast Asian Nations–funded workshops on environmental toxicology and sustainable development in Cambodia, Laos, and Myanmar in 2002 [43].

The CRI's specialist environmental toxicology unit, the International Centre for Environmental Health and Toxicology (ICEHT), was named a Centre of Excellence in Environmental and Industrial Toxicology by the United Nations Environment Programme in 1990 [44]. More significantly, the ICEHT was designated a WHO Collaborating Centre for Capacity Building and Research in Environmental Health Science in December 2005 [44,45].

**Box 1**

Roger Walk's Positions at PM and Affiliated Institutions

- Manager, Philip Morris External Research Program 2000 onwards;
- Director, PM Scientific Affairs 2003 onwards;
- Director, PM Worldwide Scientific Affairs 1999 onwards;
- First secretary of Asian Regional Tobacco Industry Scientists Team
-   (ARTIST) 1999;
- Acting Director Asia/Japan/Australia 1999 onwards;
- Research Fellow, PM Worldwide Scientific Affairs 1999;
- Research Fellow, PM Scientific Affairs, Asia-Japan-Australia,
-   (Hong Kong) 1996–1999;
- Executive Scientist, Asia, Institut für Industrielle und Biologische
-   Forschung GmbH (INBIFO) 1995–1996;
- General Manager, Contract Research Center (CRC) 1988–1995;
- Research Director, INBIFO 1985–1988;
- Scientist, and Department Head, INBIFO 1978–1985.

Source: Glossary of Names for Philip Morris USA, Inc. Privilege Log [65]. Note: Walk is also referred to as Ruediger Walk in some documents, and listed as *Walk, Ruediger A., Dr. (Roger)* in the PM Glossary of Names included in its Privileged Log.

The Institute's relationship with tobacco industry scientists assumes particular national significance from its president and founder, Professor Dr. Her Royal Highness Princess Chulabhorn Mahidol, an active scientist and the youngest daughter of Thailand's King Bhumibol Adulyadej [46].

This paper examines the means by which PM obtained access to the CRI, to its scientists and students, and to the broader Thai scientific and policy communities. It focuses in particular on how one prominent consultant has influenced the study of environmental toxicology at the CRI and has developed relations with key officials and local scientists so as to advance the interests of PM within Thailand and across Asia.

## Methods

This study is based on analysis of tobacco industry documents available in tobacco control community online collections, the Legacy Tobacco Documents Library (http://legacy.library.ucsf.edu/), which incorporates the British American Tobacco Documents Archive, operated by the University of California, San Francisco; and Tobacco Documents Online (http://tobaccodocuments.org/). Online document collections operated by US-based tobacco companies under the terms of the 1998 Master Settlement Agreement were also searched. The provenance, mechanics, and limitations of using tobacco industry documents have been described elsewhere [47–54].

The research design reflects the iterative nature of tobacco industry document research. Initial document searches focused on possible references to the Asian ETS programme in Thailand, and employed broad search terms as “Thailand”, “science”, “ETS”, and the names of high-profile individuals involved in the ETS programme such as John Rupp [21]. Documents retrieved indicated that only one recognised Thai scientist, Professor Malinee Wongphanich of Mahidol University, was recruited into the programme [19], and that leading industry consultants Roger Perry of Imperial College, London, and George Leslie, coordinator of the UK ETS consultants group Association of Research on Indoor Air [19], visited Bangkok for a series of lectures and meetings in 1993.

More significantly, these findings yielded new terms which generated further searches and resulted in the retrieval of documents detailing the relationship between PM scientists and the CRI. A total of 163 documents were retained from searches of the collections: 98 on PM scientists and the CRI (dated between 1994 and 2002); 33 on ETS and Thailand (1987–2002); 19 on Walk and Thailand's ingredients disclosure regulation (1991–1998); and 13 on Asian Regional Tobacco Industry Scientists Team (ARTIST) (1998–1999). Documents collected were analysed using validation techniques based on a hermeneutic process [55], with particular importance attached to corroboration of interpretation between authors and the triangulation of industry documents with other data sources (notably via the Internet).

On a final note related to methods used in constructing this study, the authors emphasise that because of the extreme sensitivity attached to discussion of Thailand's royal family, they decided not to consult any Thai individuals or organisations during the development of this paper. Responsibility for this study rests solely with the authors.

## Results

### Building Relationships: Roger Walk and Mathuros Ruchirawat

Initial contacts with the Thai scientific community were established by George Leslie, a key figure in the early stages of the ETS programme [56–59], and Roger Perry of Imperial College, London [21]. While documents indicate links between the two consultants and the CRI [60,61], the key individual in the development of PM's relationship with the Institute has been Dr. Roger Walk a toxicologist and biochemist who has long served PM in several capacities (see Box 1).

Walk has held leading positions at PM's research facilities, INBIFO [12], and the Contract Research Center [62], and has served as Director of Worldwide Scientific Affairs. He has also served on PM's Scientific Research Review Committee, created to ensure that research conducted or funded on tobacco or smoking by PM was serving relevant corporate needs [63], and managed its external research program that was recently described as a vehicle to “garner scientific credibility for PM” [64].

From the early 1990s, Walk's duties entailed a significant focus on Asia, including roles as research fellow at PM Scientific Affairs Hong Kong which oversaw projects in Asia, Japan, and Australia, and acting director for the region from 1999 [65]. He was involved in two key strategies to resist the growing social intolerance toward smoking: undermining independent scientific discourse on ETS, and promoting, in alliance with the hospitality industry, “accommodation” of smokers and nonsmokers in bars, restaurants, and other public settings [66].

Walk also served as secretary of ARTIST, a coalition of overseas cigarette manufacturers operating in the region that has actively sought to involve Asian tobacco monopolies and companies [67]. In this role, he oversaw implementation of strategies to support science on new technologies for improvements to indoor air quality, and promote accommodation strategies premised on “an environment where smokers and nonsmokers can comfortably coexist” [68,69].

In 1996, Walk was charged with planning the tobacco industry's response in Asia to the pending IARC report on ETS [67], and developed PM's “Plan for Asia 1996/1997.” The report identified opportunities to maintain the social acceptability of smoking in the region, to ensure favourable regulation, and to “communicate our views to, and put the issues into perspective with, opinion leaders and the public, and position PM as a reasonable partner in the decision-making process” [22].

### Walk's Activities in Thailand

Information obtained via searches of the document collections indicates that Walk's principal contact at the CRI has been Dr. Mathuros Ruchirawat, the current Vice President for Research [70], Deputy Director of the WHO Collaborating Centre, and an associate professor in the department of Pharmacology at Mahidol University in Bangkok [71]. PM documents indicate both that Mathuros has long recognised Walk's connections to the tobacco industry, and has regarded them as unproblematic. In 1993, Walk stated that “CRC's and INBIFO's affiliation with PM is known to the main coordinator (Dr Mathuros) but probably not to the other scientists” [72]. At a 1999 meeting between PM scientists and CRI officials in Bangkok, Mathuros reportedly reaffirmed her readiness to work with the tobacco industry [73].

Walk appears to have initially contacted Mathuros in October 1991 to request a meeting during a forthcoming trip to Bangkok [74]. Correspondence in early 1992 reveals that topics raised at the meeting included an upcoming CRI science congress and the institute's planned construction of an inhalation research laboratory [75]. Having informed Mathuros that he would attend the Second Princess Chulabhorn Science Congress in December 1992, Walk proposed making a “contribution to the scientific program” [75]. Following consultation with Richard Carchman [76,77], then PM's manager of biochemical research and later director of scientific affairs [65], he submitted an abstract for a poster based on INBIFO data previously presented at a May 1990 meeting of the Gesellschaft für Umwelt-Mutationsforschung [77], the German-speaking section of the European Environmental Mutagen Society.

Walk also invited the Princess, Mathuros, or “any other distinguished Thai toxicologist” [75] to inspect inhalation facilities at the CRC in Belgium and those of its “sister institute” the INBIFO in Cologne, adding that he hoped to “be of assistance to the further development of toxicology in your country” [75]. Documents indicate that Mathuros visited PM-run facilities in Europe on at least three occasions between August 1992 and July 1994 [78–82], and during the 1992 trip she and Walk discussed his support for the CRI's efforts to secure “training positions for young Thai toxicologists in Germany” [83].

Walk was also offered his first direct association with the institute in the form of consultancy work on an inhalation laboratory project [78]. His extensive correspondence with Mathuros details discussions of the project's technical specifications [84–88], and of consultancy trips to Bangkok [89–91]. Much of the cost of Walks' trips to Bangkok were underwritten by the CRI via funding from the Deutsche Gesellschaft für Technische Zusammenarbeit, a German development fund [92–95].

### Lawyers, Science, and Regulation in Southeast Asia

The critical role of lawyers in tobacco industry efforts to manipulate scientific and regulatory affairs is well established via both academic research [96–99] and litigation [100]. Walk's key contact with PM's legal division during the early 1990s appears to have been Tony Andrade of the legal firm Shook, Hardy & Bacon, a US-based law firm that has acted as counsel for the tobacco industry internationally [65,100–103]. Andrade was seconded to PM's in-house legal department in Lausanne, Switzerland from 1991 until 1995, serving as Vice President, PM Worldwide Regulatory Affairs [65].

Correspondence between Walk and Andrade frequently touched on developments at the CRI, which Walk described as “the leading institution in bioresearch and toxicology in Thailand (and probably Southeast Asia)” [72]. In July 1992, Walk informed Andrade of his planned participation in the Second Princess Chulabhorn Science Congress in November, suggesting it would “expand our relations to Thai scientists” [83]. His post-conference report depicted the congress as “an excellent forum to develop new relationships with Thai scientists, which could be useful in the future” [104], adding that he had “focussed on local Thai scientists (e.g. institute heads at the Kasetsart University, Bangkok) being involved in research and administration of food ingredients” [104].

A further source of Walk's claimed success in insinuating himself within the Thai scientific community was his consultancy work for the CRI: “[i]n this context, I am interacting not only with the key-staff of the Chulabhorn Research Institute including the Princess but also with high-ranked ministry officials being involved in the building project. I think that this support will open many doors for us” [104].

In June 1993, Walk informed Andrade of his further progress in developing “scientific contacts with CRI and other Thai scientists” over the preceding year and a half [72]. Among those Walk described as contacts were top level officials at the CRI including the Princess, Mathuros, Professor Wichit Srisa-an, the institute's Vice President, and Professor Stitaya Sirisinha, who headed the Institute's Laboratory of Immunology. Others named by Walk included two CRI scientists who were also faculty at Mahidol University, a department head from Kasetsart University, and an official from the Ministry of Public Health [72]. Walk also noted that he been “added to the external advisory board of the CRI and invited to give a course on inhalation” [72], and that his growing responsibilities were “well suited to sustain a good personal scientific relationship to Thai scientists and to be asked for advice in inhalation toxicity related issues” [72].

Such networking occurred in the context of attempts by transnational tobacco corporations to undermine Thailand's comprehensive 1992 Tobacco Products Control Act [105], which included a requirement for public disclosure of cigarette ingredients. A previous study demonstrates PM's key role in the campaign to undermine disclosure regulation [106], and Andrade served on PM's three-man “ingredients task force” that sought to coordinate a consistent response to ingredients disclosure issues internationally and across the industry [107].

PM documents indicate that Walk was also closely involved in discussions with leading PM strategists on ingredients disclosure tactics [108–114]. The two year plan for PM Scientific Affairs Asia he devised in 1996 committed the division to supporting corporate activities to oppose by-brand disclosure of ingredients in Thailand by “assisting in the timely provision of ingredient information in an appropriate context where required” [22], and a January 1992 memorandum from Andrade (“Philip Morris Counsel”) to Walk (“Philip Morris Employee”) concerned contacts with local Thai scientists regarding the ingredients disclosure legislation [115], though its contents are not publicly accessible [116].

### Walk's Contribution to Curriculum Development

In 1993 Walk was offered a teaching post on an upcoming inhalation course that was “part of the institute's international training courses in toxicology” [72]. He was subsequently offered a renewable two year contract by Mathuros in October 1993 to act as an “expert consultant in establishing biomedical research facilities and an inhalation toxicology program” [117]. Later that year, Walk sought to further expand his role within the CRI, reminding Mathuros that he was willing to contribute to “scientific activities of the CRI especially in the area of environmental toxicology and carcinogenesis” [118].

This growing involvement in curriculum development was detailed in a July 1994 letter to PM's Manager of Worldwide Regulatory Affairs Jan Goodheart in New York. Walk reported that he had been added to the international faculty on a UN-funded [82] post-graduate environmental toxicology, technology, and management programme that the CRI planned to launch in 1996 in collaboration with the Asian Institute of Technology [119,120].

He also advised Goodheart that he had been asked to organise a symposium or workshop “on a current topic of toxicology” for the CRI's international conference on environmental toxicology in 1996 [82]. The resulting one-day pre-conference workshop on 9 December 1996 focused on indoor air quality and health, and consisted of presentations by Walk, industry consultant Ragnar Rylander [12,121], and Max Eisenberg [122], director of the industry-led Center for Indoor Air Research [5,123]. Tobacco industry links of the three presenters were not listed in the day's program, nor were they in a previous CRI call for abstracts [124].

Walk's expertise was also sought by the CRI in developing other programme course units, “e.g. Environmental Risk Assessment and Management or Study Design or a seminar on specific ‘cases' in toxicology” [82]. In 1999, he was “invited to further contribute to the joint Environmental Toxicology program of the Asian Institute of Technology, CRI, and Mahidol University, Bangkok” noting that “[t]hree years ago I contributed together with a group of international experts to the development of the curriculum of this program” [125]. His participation, he suggested, would “support our core strategy of developing venues for the objective presentation of high quality scientific data” [125]. In accepting this latest appointment, Walk wrote to Mathuros suggesting that his greatest potential contributions to the program would be, “in principle, all areas of environmental and occupational toxicology” [126].

### The 1999 Symposium

A January 1999 meeting between PM scientists Walk, Mingda Zhang, and Raymond Lau, and CRI faculty Mathuros, Dr. Jutamaad Satayavivad, and Dr. Amniporn illustrates the scope of engagement with toxicology research that the CRI provided the tobacco corporation. In expressing “the institution's appreciation” [73] for Walk's consultancy work and his contribution to the postgraduate curriculum, Mathuros added that the institute “would welcome opportunities to corporate [sic] with the Industry on scientific issues. Such corporations [sic] will be evaluated on its scientific merits and should not be affected by the political issues associated with the industry in some environments” [73].

Specifically, she asked PM to “corporate [sic] and support research in the area of indoor air exposure assessment in Thailand,” and encouraged Walk “or other scientists from PM to become visiting professors of the Institute and give lectures to students in the postgraduate program in toxicology” [73]. Mathuros also requested “Walk's assistance and PM's support in organizing a half-day workshop on indoor air quality” [73], as part of the Fourth Princess Chulabhorn Science Congress. This last request was apparently based on the success of the 1996 pre-conference scientific workshop described above [73].

The result was Symposium 10 of this Congress, “Indoor Air Quality,” on 1 December 1999. Chaired by Walk and Dr. Elizabeth Anderson of Sciences International USA, and formerly of the US Environmental Protection Agency [127–130], the line-up of speakers included Walk, PM's Raymond Lau, Dr. Keith Phillips of Covance Laboratories UK, and Anderson. Philips had previously undertaken research projects commissioned by the tobacco industry [131], while Anderson had acted as a consultant to tobacco companies challenging US EPA proposals on pesticide regulation [132,133]. PM funded Walk and Lau's travel expenses, and contributed US$12,000 toward the expenses of other speakers including Anderson and Phillips [134]. Mathuros wrote on behalf of the CRI and the Congress organising committee to “thank Philip Morris USA for this generous support” [127].

As described by Walk, overseeing the symposium presented PM with an opportunity to “develop a venue for the objective publication and presentation of high-quality scientific results (credibility core strategy)” and to “provide quality information on technical solutions in support of accommodation” [125].

Such opportunities represented “core strategies of WSA [PM Worldwide Scientific Affairs] and are fully integrated into our business goals” [125]. Proceedings for the congress were published in 1999 [135] but are not available via accessible tobacco industry document collections or other online sources.

### Benefits of the CRI Connection to PM

The value of Walk's relationship with the CRI has been his ability to establish contacts at the institute, within the wider Thai scientific community and, particularly, to influence curriculum design on toxicology. Importantly, the significance of this association extended beyond Thailand: “[i]n summary: The CRI takes a leading position in the training and development of local manpower in toxicology in the Pacific. Their focus during the next 5 years is in training of experts who can serve as regulators at government agencies and their scientific advisors…To contribute to these training efforts will significantly help to ensure scientific objectivity and enforce the views that local problems in these countries cannot always be solved by adopting ‘solutions' from the US or Europe” [82].

Further evidence of the value of the CRI connection to PM is provided by a short 1999 report, “How have interactions with regulators and scientists furthered our objectives?” in which Walk highlighted four key contributions under the heading Credibility and Accommodation of Smokers and Nonsmokers Core Strategies: “Invitation to organize Indoor Air Quality Seminar at Princess Chulabhorn Congress, Bangkok: Example for development of venues for publication”; “Continuous support of Executive VP for Scientific Research [Mathuros] with public quality science information, presenting research data at previous scientific conferences, execution of contract projects with scientific excellence”; “Invitation to contribute as lecturer to toxicology postgraduate training program in Thailand: Example for development of venues for publication and presentation”; “Contribution to development of training curriculum as expert in inhalation toxicology” [136].

### Postscript: The International Centre for Environmental Health and Toxicology

The Web site of the ICEHT, the CRI's specialist toxicology unit, demonstrates that tobacco industry consultants continue to exert influence on the work of the institute. Walk is currently (at the time of this writing) cited amongst visiting faculty on the ICEHT Inter-University Post-Graduate Education Program on Environmental Toxicology, Technology and Management, and is described as Roger Walk, Ph.D. (Biology) from Ruhr-University Bochum, Germany [137]. His affiliation on the announcement for an August 2006 post-graduate course in Environmental Toxicology, Technology and Management, however, is given as “Director, Worldwide Scientific Affairs PM, USA AND Acting Director Asia-Japan-Australia (Richmond VA) USA” [138]. He is also listed as visiting faculty on Mahidol University's Environmental Science, Technology and Management Web site, where his affiliation is given as Worldwide Scientific Affairs PM, USA [139].

The Web site also reveals the roles of Donald J. Ecobichon and J. F. Borzelleca, both of whom have long-standing ties to the tobacco industry [140,141], in teaching at the ICEHT. Ecobichon, who helped organise the tobacco industry-sponsored 1989 McGill ETS symposium [142–144], was amongst the faculty on the CRI's training course on environmental and industrial toxicology in 1991 [145] and 1994 [146]; on the joint CRI-Asian Institute of Technology course in 1995 [147]; and on a series of workshops on environmental toxicology and sustainable development held in Cambodia, Laos, and Myanmar in 2002 [43]. He is currently (at the time of this writing) listed as visiting faculty on the ICEHT Web site [137], as an expert advisor on the institute's internet-based information exchange service [148], and has contributed to *Introduction to Environmental Toxicology* [149], a set of training modules used at the institute. Borzelleca, emeritus professor at Virginia Commonwealth University, has worked as an outside consultant with the American Tobacco Company and PM since 1980 [141] and edits the journal *Food and Chemical Toxicology,* which has published studies by a number of tobacco company scientists [150]. His association with the CRI is similar to Ecobichon's, having taught on a number of training courses [145,146,151] and contributed to *Introduction to Environmental Toxicology* [149].

The 2005 designation of ICEHT as a WHO Collaborating Centre for Capacity Building and Research in Environmental Health Science is the most significant example from a broad range of connections between CRI and WHO. The institute is an approved institution for WHO staff fellowship placements [152], and its toxicology research facility is listed as a Sustainable Development and Healthy Environments Collaborating Centre by the WHO South-East Asian Regional Office (SEARO) [153]. In 2002, the CRI hosted WHO's International Conference on Environmental Threats to the Health of Children [154], and in 2003 organised a workshop on children's health and the environment in cooperation with the WHO and its SEARO and Western Pacific Regional Office regional agencies [155].

There has also been extensive collaboration on environmental issues. The institute collaborated with WHO's Sustainable Development and Healthy Environments Cluster in organising a December 2004 meeting in Geneva; a key outcome was an agreement between Princess Chulabhorn, the WHO, and the WHO Kobe Centre to cooperate on priority research into environmental health issues [156]. In 2005 and 2007, the institute co-hosted ministerial regional forums on environment and health in collaboration with WHO, the Asian Development Bank, and the United Nations Environment Programme [157,158].

In the field of chemical safety, the Princess is a recent recipient of WHO's Intergovernmental Forum on Chemical Safety agency's special recognition award [159]. CRI Vice President for Research Mathuros is a member of the advisory group of WHO's task group on environmental health risks to children [160], and has represented SEARO at recent steering committee meetings of WHO's International Programme on Chemical Safety [161,162].

## Discussion

The documents analysed above demonstrate how tobacco industry scientists have established durable relationships with the CRI, a key regional centre of scientific training and expertise in Thailand. The royal connection makes any critical discussion of the institute within Thailand an extremely delicate matter. The King commands enormous public respect and affection, and is regarded as a semi-divine figure [163,164]. Public criticism of the monarchy remains off-limits, not least because the Thai state has aggressively used lèse-majesté - the proscription of any perceived insults to the monarchy - to protect the crown [165–167]. This prohibition has resulted in a near total absence of any critical analysis of the royal family.

The establishment of close links with the CRI not only advances industry strategies to influence scientific research and debate around tobacco and health, particularly regarding ETS, but allows it to form links to academic institutions [168–170] and to build relationships with national elites [171–173]. Such strategies are familiar in North and South America and in Europe, but assume particular significance in the national and regional contexts presented here.

The importance attached by tobacco companies to developing links with research institutions and individual scientists cannot be understood in isolation from broader policy debates. Gaining access to the Thai scientific community assumed heightened significance in the context of Thailand's comprehensive 1992 Tobacco Products Control Act, which included a requirement for public disclosure of cigarette ingredients [105]. Transnational tobacco corporations operating in Thailand mounted an aggressive challenge to the disclosure clause [106], diluting it to the extent that a leading advocate of the Act has described the final version of the ingredients regulation as “useless” [174].

More broadly, the documents reviewed above indicate that this association with the CRI served to promote PM's regional interests via interactions with faculty, government officials, and local scientists and provided important opportunities to advance industry arguments in symposia and publications. The organisation of sponsored symposia on ETS at CRI as elsewhere in Southeast Asia [20] has been particularly important to tobacco corporations. It has been previously demonstrated that proceedings of such symposia can have an important policy impact “because they are often cited as if they are peer-reviewed articles, as if they are balanced reviews of the scientific literature, and with no disclosure of their industry sponsorship” [175]. Furthermore, the active and ongoing involvement of industry consultants in curriculum development and the training of future researchers and regulators is particularly disturbing and, in our view, wholly inappropriate.

Thailand's political system places a particular premium on securing close relationships with key individuals, and indeed the successes of the country's remarkable tobacco control movement owe much to the personal connections of key health advocates [176]. It is clearly difficult to assess the validity or consequences of Walk's claim to have developed links with “high-ranked ministry officials” [104] but the achievement of a clear, albeit indirect, link with Thailand's powerful and revered royal family is of real national significance.

The family's historic interest in health issues is evident in King Bhumibol's 1992 establishment of the Prince Mahidol Award, conferred annually upon individuals or institutions which have demonstrated outstanding international contributions to advancing medical and public health services [177]. Previous awardees include leading tobacco control researchers Sir Richard Doll, Sir Richard Peto, and Jonathan Samet, as well as Margaret Chan, the current Director General of WHO. The association of a research institute that enjoys royal patronage with the world's largest cigarette corporation contrasts starkly with Prince Mahidol's status as the “father of modern medicine and public health of Thailand” [177].

From an international perspective, particular concern is raised by the CRI's recently awarded status as WHO Collaborating Centre for Capacity Building and Research in Environmental Health Science with the responsibility to “promote & assist capacity building activities in Environmental Health Toxicology & Risk Assessment” [178]. The persistent involvement of industry consultants in CRI activities described above is hard to reconcile with the recommendations of the Zeltner committee inquiry into tobacco industry influence within WHO in 2000 [13]. These recommendations sought, inter alia, clarification, strengthening and expansion of rules against conflict of interest involving the tobacco industry; additional safeguards against industry efforts to distort scientific research associated with WHO and affiliated organizations; and countering of industry tactics to oppose tobacco control in developing countries [179].

The committee also clarified that its recommendations to protect the integrity of WHO's decision making were “intended for application throughout WHO, including within its Collaborating Centers” [13]. It is not clear whether the relationships described above directly contravene the recommendations or revised WHO practices, but their persistence suggests the need to strengthen WHO's response to the committee's recommended “steps to educate their scientific investigators and collaborators about tobacco company efforts to undermine research” [180].

The director of WHO's Tobacco Free Initiative was recently reported as stating that the organisation had placed “a complete firewall” between itself and tobacco companies [181]. The links between PM scientists and a WHO Collaborating Centre outlined here suggest that this firewall is not impenetrable, and highlight a much broader challenge posed to international tobacco control efforts, notably with respect to Article 5.3 of the WHO Framework Convention on Tobacco Control, which addresses the need to protect public health policies from the vested interests of the tobacco industry [182].

Questions around inadequate oversight of conflicts of interest are echoed in wider concerns about WHO relations with the private sector [183–186] while WHO monitoring of Collaborating Centres was subject to severe criticism in the context of influenza vaccine development [187]. Since the network of WHO Collaborating Centres rests on the principle of “using national institutions for international purposes” [188], the documents presented above suggest that more rigorous safeguards are required to ensure that such use advances public health goals rather than the objectives of transnational corporations.

## Abbreviations

ARTIST: Asian Regional Tobacco Industry Scientists Team
CRI: Chulabhorn Research Institute
ETS: environmental tobacco smoke
IARC: International Agency for Research on Cancer
ICEHT: International Centre for Environmental Health and Toxicology
INBIFO: Institut für Industrielle und Biologische Forschung
PM: Philip Morris
SEARO: [WHO] South-East Asian Regional Office
WHO: World Health Organization
